# A hypoenergetic diet with decreased protein intake does not reduce lean body mass in trained females

**DOI:** 10.1007/s00421-020-04555-7

**Published:** 2020-12-01

**Authors:** Alice G. Pearson, Lee Alexander, Oliver C. Witard, Thomas E. Coughlin, Kevin D. Tipton, Ian H. Walshe

**Affiliations:** 1 Department of Sport and Exercise Sciences, Durham University, Durham, UK; 2 Physiology, Exercise and Nutrition Research Group, University of Stirling, Stirling, UK; 3 Centre of Human and Applied Physiological Research, King’s College London, London, UK; 4 Department of Sport, Exercise and Rehabilitation, Northumbria University, Newcastle, UK

**Keywords:** Energy restriction, Weight loss, Body composition, Diet composition

## Abstract

**Purpose:**

Increasing protein intake during energy restriction (ER) attenuates lean body mass (LBM) loss in trained males. However, whether this relationship exists in trained females is unknown. This study examined the impact of higher compared to lower protein intakes (35% versus 15% of energy intake) on body composition in trained females during 2 weeks of severe ER.

**Methods:**

Eighteen well-trained females completed a 1-week energy balanced diet (HD100), followed by a 2-week hypoenergetic (40% ER) diet (HD60). During HD60, participants consumed either a high protein (HP; 35% protein, 15% fat) or lower protein (CON; 15% protein, 35% fat) diet. Body composition, peak power, leg strength, sprint time, and anaerobic endurance were assessed at baseline, pre-HD60, and post-HD60.

**Results:**

Absolute protein intake was reduced during HD60 in the CON group (from 1.6 to 0.9 g·d·kgBM^−1^) and maintained in the HP group (~ 1.7 g·d·kgBM^−1^). CON and HP groups decreased body mass equally during HD60 (− 1.0 ± 1.1 kg; *p* = 0.026 and − 1.1 ± 0.7 kg; *p* = 0.002, respectively) and maintained LBM. There were no interactions between time point and dietary condition on exercise performance.

**Conclusion:**

The preservation of LBM during HD60, irrespective of whether absolute protein intake is maintained or reduced, contrasts with findings in trained males. In trained females, the relationship between absolute protein intake and LBM change during ER warrants further investigation. Future recommendations for protein intake during ER should be expressed relative to body mass, not total energy intake, in trained females.

## Introduction

Many athletes employ weight loss strategies to enhance body composition and achieve health or performance goals. In particular, weight class sports, such as mixed-martial-arts and weightlifting (Barley et al. 2018; da Silva Santos et al. 2016; Matthews and Nicholas 2017), or those with an aesthetic component, such as body building and gymnastics (Bloodworth et al. 2017; Fagerberg 2018), typically necessitate body composition modification around training and competition. Athletes may achieve their desired body composition through a reduction in energy intake while maintaining or increasing energy expenditure. Hypoenergetic diets have demonstrated efficacy as a weight loss strategy in athletic populations. However, the desired loss of fat mass (FM) is often concomitant with a reduction in lean body mass (LBM) (Pons et al. 2018; Willoughby et al. 2018), which can be detrimental to athletic performance (Lalia et al. 2018). Therefore, weight loss strategies that attenuate the loss of LBM have been investigated.

One such strategy that has demonstrated efficacy in ameliorating LBM loss during weight loss is the manipulation of dietary protein intake. Exogenous protein supply is required to stimulate muscle protein synthesis (MPS) and induce positive nitrogen balance (Witard et al. 2014). Frequent stimulation of MPS by protein consumption helps maintain LBM. However, during periods of energy restriction, net nitrogen balance and rates of protein turnover are perturbed (Carbone et al. 2019), which can lead to the loss of LBM. Short periods of moderate energy restriction may increase basal rates of muscle protein breakdown (MPB) by up to 60% relative to energy balance (Carbone et al. 2014). Although, it is currently accepted that LBM loss during energy restriction is attributed to a predominant reduction in MPS (Hector et al. 2018; Pasiakos et al. 2013). It has been demonstrated that increasing dietary protein intake during energy restriction can increase rates of MPS (Hector et al. 2018) and attenuate the loss of LBM.

Previous weight loss interventions, ranging in duration from 2 to 20 weeks, have demonstrated a preservation of LBM with 1.2–2.4 g·d·kgbody mass^−1^ (BM) protein consumption in untrained and overweight individuals (Gordon et al. 2008; Hector et al. 2018; Layman et al. 2005; Leidy et al. 2007; Longland et al. 2016; Pasiakos et al. 2013). However, a paucity of data exist on the impact of increased dietary protein intake during hypoenergetic weight loss in trained individuals. Walberg and colleagues reported a retention of positive nitrogen balance when total dietary protein intake was increased to 1.6 g·d·kgBM^−1^ vs. a daily protein intake of 0.8 g·d·kgBM^−1^ among male weightlifters (Walberg et al. 1988). Although, increased provision of exogenous amino acids does not necessarily translate to LBM change (Mourier et al. 1997). Mettler et al. (2010) previously demonstrated a significant amelioration of LBM loss in trained males when a 2-week hypoenergetic diet contained 35% of energy intake from protein (2.4 g·d·kgBM^−1^), relative to 15% (1.0 g·d·kgBM^−1^). Although Mettler did not measure rates of MPS, these data support those previously reported in untrained populations (Gordon et al. 2008; Hector et al. 2018; Layman et al. 2005; Leidy et al. 2007; Longland et al. 2016; Pasiakos et al. 2013) and highlight the importance of protein intake during weight loss in athletes. However, it is unknown whether sex differences would permit the previous findings in trained males (Mettler et al. 2010) being translated to trained females. There is evidence that resistance-trained females consuming higher protein intakes increase LBM significantly more so than those consuming lower protein intakes during energy balance (Campbell et al. 2018). Nonetheless, no study to date has investigated the effect of the protein composition of hypoenergetic diets on body composition and various exercise performance parameters (strength, speed, endurance, and power) in well-trained females.

Apparent sex differences in human musculature exist (Jahn et al. 1999), which may result in varying metabolic and body composition outcomes during energy restriction. During energy balance, there are no apparent sex differences in basal MPS (Dreyer et al. 2010; Fujita et al. 2007), or the acute post-prandial or post-exercise MPS response (Dreyer et al. 2010; West et al. 2012), when corrected for skeletal muscle mass in young adults. Nonetheless, the apparent lack of sex-based differences in MPS may be attributed to the short duration of these studies. Potentially, extended time periods (i.e., training) are required to detect sex differences in muscular adaptations. Consistent with this notion, Scalzo et al. (2014) utilised a ^2^H_2_O-based method to estimate integrated MPS over a 4-week period with sprint interval training and reported higher rates of MPS in mixed and cytoplasmic muscle fractions in males, relative to females. This study demonstrates that sex-based variations in MPS may be detected over extended periods, eventually influencing changes in LBM. This notion has been supported in untrained individuals, with males losing a greater proportion of LBM than their female counterparts during a period of energy restriction (Evans et al. 2012; Sartorio et al. 2005). Taken together, these data suggest that metabolic and body composition responses to energy restriction likely differ between well-trained males and females, and thus extrapolation of the previous findings (Mettler et al. 2010) to trained females may not be appropriate.

Therefore, the aim of the present study was to investigate whether higher compared to lower protein intakes attenuate the loss of LBM during energy-restricted weight loss in well-trained females. We hypothesised that increasing dietary protein intake relative to total energy intake would ameliorate reductions in LBM during 2 weeks of severe energy restriction.

## Methods

### Subjects

Twenty-four trained females representing a range of sports (football, netball, rowing, Muay Thai, and athletics) volunteered to participate in the study after providing informed written consent. Inclusion criteria for the study were female competitive athletes aged 18–35 years, completing at least three training sessions per week. Participants with body fat within the healthy range of 15–30% and with a regular menstrual cycle were included. All types of oral contraceptives were permitted. Participants were excluded from the study if they consumed more than 20% protein (including protein supplements) in their habitual diet.

Four participants did not meet the inclusion criteria (≤ 15% body fat; ≥ 20% habitual protein intake; and/or ≤ 3 training sessions per week). Two participants were excluded due to lack of dietary adherence during the intervention. Eighteen participants (age; 21.2 ± 3.3 years, height; 171.5 ± 6.8 cm, BM; 67.2 ± 10.0 kg, body fat; 27.2 ± 5.3%, training; 9.9 ± 2.8 h wk^−1^) completed the study. The study was approved by the School of Sport Research Ethics Committee (SSREC) at the University of Stirling and the East of Scotland NHS Research Ethics Committee (NHSREC).

### Experimental design

**Fig. 1:**
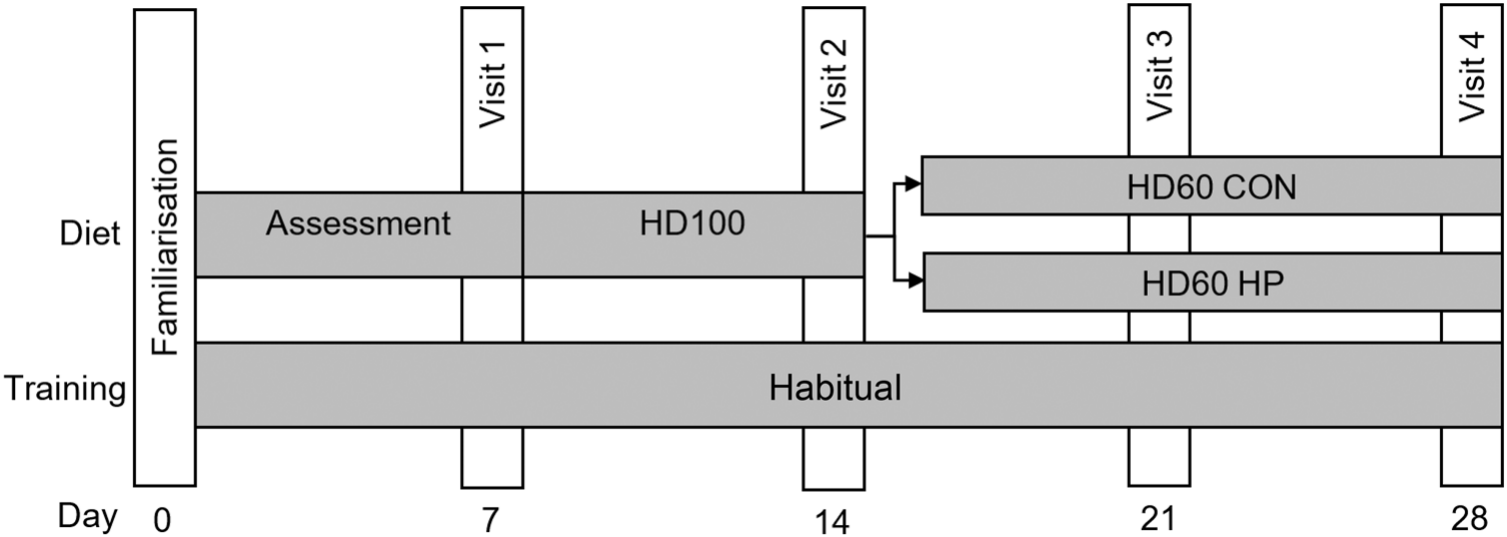
Schematic overview of study protocol. *Familiarisation* body composition and exercise protocols, *HD100* habitual diet at 100% of individual energy requirement, *HD60* habitual diet at 60% of individual energy requirement, *CON* control group (0.86 g·d·kgBM^−1^protein) at 60% of individual energy requirement, *HP* high protein group (1.71 g·d·kgBM^−1^protein) at 60% of individual energy requirement

In a parallel group design, each participant completed a familiarisation trial followed by four experimental trials consisting of body composition measurement and exercise performance tests. Participants were assigned to either a high protein (HP) (*n* = 9) or a lower protein control group (CON) (*n* = 9). The overview of the study design is shown in Fig. 1.

Participants arrived at the laboratory before 9:00 am for each experimental trial following an overnight fast (morning consumption of 500 mL water) and abstinence from vigorous exercise during the prior 24 h. Participants provided a urine sample, then height, BM, and body composition were measured in light clothing. The initial screening visit was used to obtain preliminary measures of general health (absence of illness, chronic disease, musculoskeletal disorder, and pregnancy), baseline body composition, and familiarisation of the exercise protocols. Following the familiarisation visit, participants were instructed to complete a weighed intake food diary over three separate days; a rest day, training day, and competition day, from which habitual energy intake was estimated using dietary analysis software (Microdiet V2).

During the second visit, baseline exercise performance tests were completed, and participants were prescribed a diet providing 100% of habitual energy intake (HD100) (48% carbohydrate, 33% fat, 19% protein), based on self-reported dietary intake data. Following 7 days of diet completion, participants attended the third testing session, where pre-measures of BM, body composition, and exercise performance were obtained. Participants were then prescribed a diet providing 60% of habitual energy intake (HD60) and allocated to their intervention group (HP or CON). Group allocation was randomly assigned for the first nine participants and matched for training load and anthropometric characteristics for the remaining nine participants. Following 14 days of HD60, participants returned to the laboratory for the final testing session. Post-measures of BM, body composition, and exercise performance were obtained to evaluate any diet effects.

### Dietary intervention

During HD100 and HD60, participants were provided with all food and prohibited from consuming non-prescribed food and drink, aside from the ad libitum consumption of water. The CON diet had a composition of 15% protein, 35% fat, and 50% carbohydrate, and the HP diet had a composition of 35% protein, 15% fat, and 50% carbohydrate. The energy and macronutrient intake of both groups is displayed in Table 1.

**Table 1:** Macronutrient composition of prescribed diets

	Diet	Control (*n* = 9)	High protein (*n* = 9)	*P* value
Energy (Kcals)	HD100 HD60	2114 ± 517 1347 ± 264	2173 ± 343 1302 ± 209	0.84 0.64
CHO (g) (g d^−1^ kgBM^−1^)	HD100	283 ± 54 (4.32 ± 1.26) 176 ± 32 (2.68 ± 0.78)	272 ± 46 (4.15 ± 0.83) 168 ± 26 (2.58 ± 0.49)	0.65 (0.73) 0.58 (0.74)
	HD60			
FAT (g) (g d^−1^ kgBM^−1^)	HD100	83 ± 31 (1.27 ± 0.61) 51 ± 13 (0.77 ± 0.26)	79 ± 21 (1.20 ± 0.30) 24 ± 5 (0.38 ± 0.09)	0.76 (0.74) < 0.01* (< 0.01*)
	HD60			
PRO (g) (g d^−1^ kgBM^−1^)	HD100	101 ± 25 (1.56 ± 0.55) 56 ± 9 (0.86 ± 0.23)	108 ± 25 (1.65 ± 0.41) 112 ± 18 (1.71 ± 0.31)	0.59 (0.69) < 0.01* (< 0.01*)
	HD60			

Values reported as mean ± standard deviation

*CHO* carbohydrate, *PRO* protein, *g d*^*−1*^* kgBM*^*−1*^ grams per day of protein per kilogram of body mass

*Denotes statistically significant difference between groups (*p* < 0.05; independent samples *t* test)

Protein intake was evenly distributed throughout the day with 3 meals and 3 snacks (including protein-based snacks post-exercise and pre-sleep). The diet was modified if participants disliked certain prescribed foods in attempt to enhance adherence. Group allocation was not disclosed to the participants. Participants received an additional food log diary and electronic scales during the HD60 intervention to report any other drinks or foods that were consumed or any prescribed food that was not consumed. Participants were asked to report back to investigators immediately so that the macronutrient composition could be modified accordingly the following day.

### Assessment of body composition

A whole-body dual energy X-ray absorptiometry (DXA) (Lunar, GE Healthcare Prodigy, GE Healthcare, Buckinghamshire, UK) scan was used to determine body composition at visits 1 (Baseline), 3 (Pre), and 4 (Post). This model of DXA has a coefficient of variation of 1–2% (Toombs et al. 2012). The best practice procedures, as presented by Nana and colleagues, were followed (Nana et al. 2015). In addition, participants were instructed to consume 500 mL of water upon waking to ensure a euhydrated state, which is recommended under the best practice for DXA measurement (Rodriguez-Sanchez and Galloway 2015). Participants were asked to wear minimal clothing consisting of an unwired sports bra and a pair of shorts and were asked to wear the same clothes at each subsequent testing visit. A pregnancy test was completed before the scan to ensure no participants were pregnant. The participant positioning during the scan was standardised and maintained throughout the duration of the study. Total BM, FM, LBM and body fat (BF) percentage were measured. Following body composition assessment, participants completed several exercise performance tests.

### Exercise performance

All participants took part in a familiarisation visit which included the battery of exercise performance tests undertaken in the present study. The visit included a full explanation of the procedures, with participants performing the full exercise testing protocols. Participants were instructed to record all exercise sessions completed and to maintain a similar training schedule throughout the duration of the study while ensuring abstinence from vigorous exercise in the 24 h period prior to laboratory visits. During the laboratory visits, participants completed a 5-min standardised warm-up followed by a 6 s Wingate test, a maximal isokinetic leg strength test, a 20 m sprint, and the Yo-Yo anaerobic endurance test. All machine settings were determined during visit 1 and remained constant throughout the study for each participant.

### Wingate test

A 5-min warm-up was completed on a cycle ergometer (Excalibur Sport; Lode, Netherlands). The warmup included 5ˣ5 sec sprints at sub-maximal intensity during the final 2 min before moving onto the testing ergometer. The 6 s Wingate test was performed on an electronic ergometer (Excalibur Sport; Lode) using the official Wingate software (Wingate version 1.0.13; Lode, Netherlands) and recorded maximal anaerobic power output and peak power. Prior to the test, participants completed a 1 min lead-in to increase cadence from 70 to 100 revs min^−1^. Participants were then encouraged to pedal at maximal effort, maintaining the highest possible cadence for the full 6 s. A 5 min cool down was then performed on the cycle ergometer and a ~ 20 min rest period was awarded before commencing the isokinetic strength test. A standard error of measurement of 4–6% for peak power output for females has previously been reported (Kavaliauskas and Phillips 2016).

### Isokinetic strength

Participants completed an isokinetic peak force test on the electromechanical Kin Com 125 isokinetic dynamometer (Chattanooga Group, Inc. Tennessee). Participants were seated in the testing chair with 90° of hip and knee flexion and secured into the seat through stabilisation straps. The axis of the dynamometer was then aligned with the anatomical axis of the knee joint for each participant. The dominant leg was used for testing and was assessed from 20° knee flexion to 70° knee flexion. Five submaximal warm-up muscle contractions of 2–4 s were performed; two at 50%, two at 70%, and one at 90% of participants’ perceived maximal contraction, each separated by 30 s of rest. The testing protocol consisted of at least three maximal contractions of 60°s^−1^ and 120°s^−1^. Each maximal concentric contraction was followed by a maximal eccentric contraction separated by a 5 s pause. Following a 30 s interval, the next maximal contraction was completed. The highest concentric and eccentric scores were accepted as the maximal voluntary contraction. Participants were allowed a 2 min rest interval before testing began at 120°s^−1^. Participants were vocally encouraged throughout the trials and were instructed to grip the sides of the testing seat for comfort. Approximately 15–20 min of rest occurred before the 20 m sprint test. Previous authors have reported 6.6–11% variation in performance outcomes for concentric contractions and 8.9–11.9% variation for eccentric contraction in females (Li et al. 1996).

### Speed and Yo-Yo endurance test

The 20 m sprint and Yo-Yo endurance tests were completed on hard tennis courts. Participants completed a 5 min dynamic warm-up of choice that was repeated for each trial. In the final 2 min of warm-up, participants completed 5 submaximal 20 m sprints; two at 50%, two at 70%, and one at 90% of perceived maximum speed. Two mins of rest were allocated before participants began the maximal 20 m sprint testing. Each participant completed three maximal sprints separated by 1 min of active recovery. Sprint time was recorded through electronic speed gates (Brower Timing, Draper, USA) set at 0, 5, and 20 m. Participants were set 1 m behind the timing gates and were encouraged to sprint maximally through the final gates at 20 m. The coefficient of variation of 5 m and 20 m sprints has been shown to be 3.2% and 2.9%, respectively (Altmann et al. 2015; Shalfawi et al. 2012).

Following sufficient rest (10–15 min), participants began the Yo-Yo level 1 recovery test, as previously reported (Gravina et al., 2017). When signalled by the recorded beep from the CD, participants ran to and from a cone placed 20 m from the starting point. A period of 10 s active recovery separated each 40 m shuttle. The test was terminated when the participant failed to touch the starting line in time of the beep on two continuous shuttles. All testing conditions remained consistent and each participant completed the test individually to remove the element of competition. The reliability of the Yo-Yo level 1 endurance test has been examined and shown a CV of 4.9% with performance outcomes of the test (Krustrup, 2003).

### Blood sampling

Blood samples were collected from an antecubital vein into three 5 mL vacutainers containing ethylenediaminetetraacetic acid (EDTA), serum (untreated), and lithium heparin (LH). Blood samples were centrifuged at 4°, 3500 rpm for 10 min within 2 h of collection. Serum stores were transferred into 1 mL containers and stored at − 80 °C until analysis of serum progesterone concentration. Serum progesterone concentrations were determined by a commercially available ELISA kit (Eagle Biosciences). Participants were estimated to be in the follicular phase when serum progesterone concentration was < 5 ng·mL^−1^ and in the luteal phase when ≥ 5 ng·mL^−1^ (Shaaban and Klopper 1973). A progesterone concentration range to indicate ovulation (typically 5–7 ng mL^−1^) was not used as this range would be entered both during periovulation and when leaving the luteal phase (i.e., pre-menses) and cannot be distinguished without another confirmation of ovulation, e.g., luteinising hormone concentration in urine.

### Statistical analysis

All assumptions for statistical models were assessed. Data that violated the assumptions were logarithmically transformed before statistical analysis. Statistical analysis was performed on IBM SPSS (version 25, SPSS Inc., Chicago, IL). A two-way mixed design ANOVA was used to analyse all body composition and exercise performance measures between diet conditions (CON and HP) and within time points (pre- and post- HD60). Independent *t* tests with Bonferroni corrections were used to compare time-points between conditions for all body composition and exercise performance measures. Paired *t* tests were used to detect any significant within-group changes between time points. Based on a statistical power test using Minitab 18.1 software, an estimated sample size of 17 was required to have 80% power to detect a difference in BM of 1 kg, assuming a standard deviation of 1. Thus, the acquired sample of *n* = 18 is sufficiently powered when using a dependent *t* test with a 0.05 two-sided significance level. *P* < 0.05 was considered statistically significant*.* Confidence intervals assume 95% confidence in the range of the mean. All data are reported as mean ± standard deviation (SD) unless otherwise stated.

## Results

### Dietary intake

Dietary intake data are displayed in Table 1. Energy intake was similar between HP and CON groups during HD60, and the reduction in energy intake from HD100 was achieved through a decrease in daily carbohydrate and fat intake. During HD60, mean daily protein intake in the HP group was twice the amount of the CON group. Whereas the HP group increased mean daily protein intake by 4%, the CON group reduced their protein intake by 45% from HD100 to HD60. The reduction in fat intake was more pronounced in the HP group, relative to the CON group, to allow for a greater protein intake while matching total energy intake.

### Body composition

**Fig. 2:**
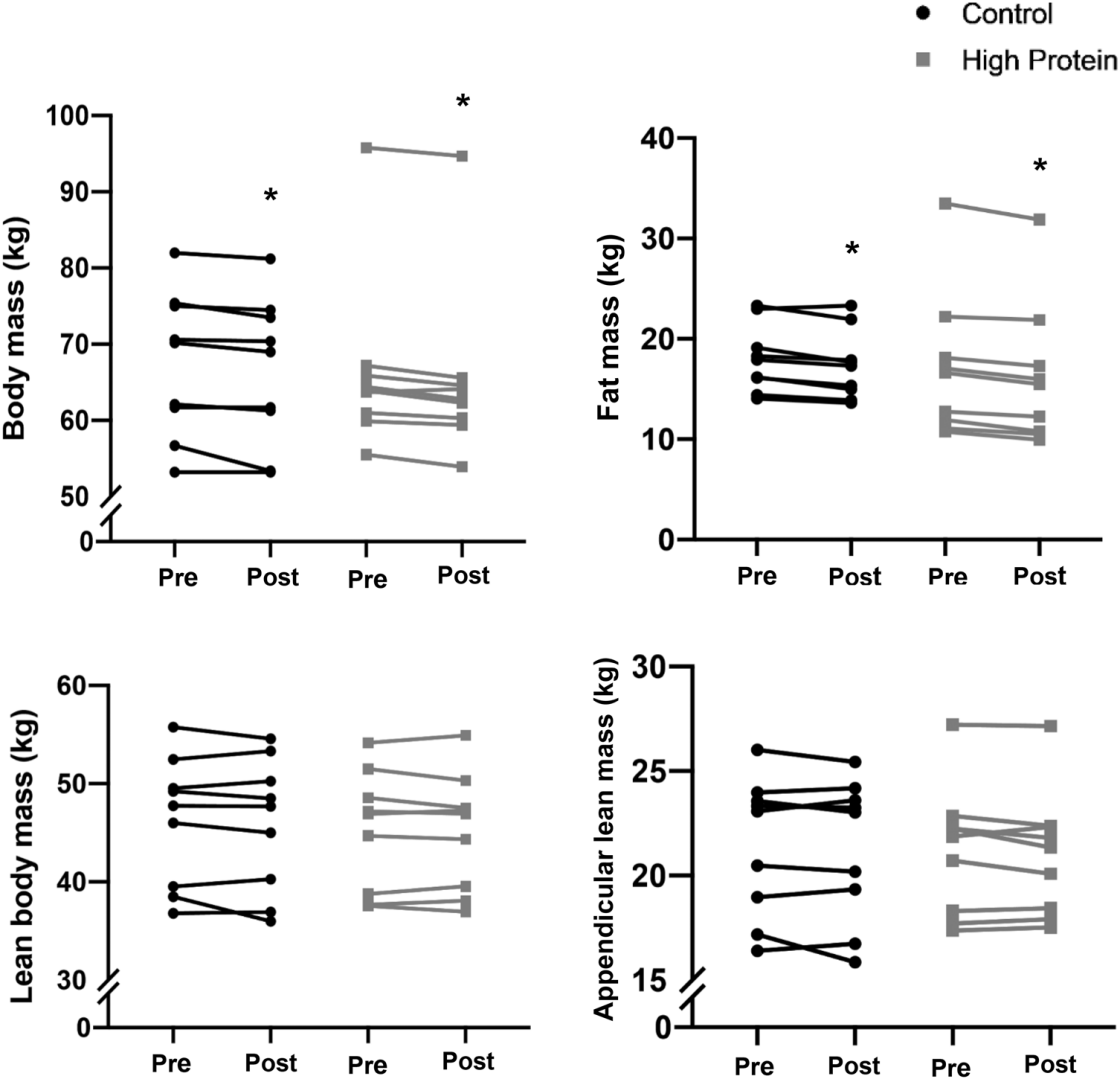
Individual body composition data at pre- and post-energy restriction. Control diet = 0.86 g·d·kgBM^−1^protein; high protein = 1.7 g.g·d·kgBM^−1^protein; pre = pre 40% energy restriction (HD60); post = post HD60. *Denotes statistically significant change from pre (*p* < 0.05; paired samples *t* test)

Total BM decreased during HD60 in both the CON and HP groups (− 1.0 ± 1.1 kg; 95% CI− 1.72 to − 0.28; *p* = 0.026 and − 1.1 ± 0.7 kg; 95% CI− 1.56 to − 0.64; *p* = 0.002, respectively); however, the difference between dietary conditions was not statistically significant. There were no differences in any body composition measurements between groups at baseline or pre-HD60 and paired samples *t* test revealed no change in body composition from baseline to pre-HD60 (all *p* > 0.05). Individual changes in body composition measurements throughout HD60 are displayed in Fig. 2. Overall, there were no significant interactions between time point (pre-post) and diet group on BM, FM, LBM, or appendicular lean mass (ALM). The loss of FM was slightly greater in the HP group (− 0.9 ± 0.4 kg; 95% CI − 1.14 to − 0.63; *p* < 0.001) than the CON group (− 0.7 ± 0.6 kg; 95% CI − 1.08 to − 0.34; *p* = 0.005) from pre-post HD60, though there was no between-group difference (*p* = 0.451). During HD60, absolute LBM did not change in either the HP or CON group (− 0.1 ± 0.7 kg; 95% CI − 0.6 to 0.4 and − 0.3 ± 1.1 kg; 95% CI − 1.1 to 0.4, respectively), with no between-group differences (*p* = 0.627). Likewise, the relative LBM change from pre- to post- HD60 was not significantly different between groups (*p* = 0.563; HP: − 0.23%; 95% CI − 1.5 to 1.0 kg and CON: − 0.74%; 95% CI − 2.8 to 1.3 kg). Notably, one participant lost 6.6% of LBM during HD60, though even when data were analysed with omission of this outlier, the between-group difference remained insignificant.

### Exercise performance

#### HD100 (baseline-pre) differences

There were no significant differences in exercise performance between dietary groups at baseline. The CON group experienced a statistically significant increase in isokinetic concentric strength at both 60°s^−1^ (*p* = 0.01) and 120°s^−1^ (*p* = 0.01) from baseline to pre-HD60. No significant changes from baseline to pre-HD60 were found in the HP group for isokinetic concentric strength or in either dietary condition for isokinetic eccentric strength at 60°s^−1^ or 120°s^−1^. Yo-Yo test scores improved in the HP group from baseline to pre-HD60 (*p* = 0.042) but remained constant in the CON group. Neither group experienced change in 5 m nor 20 m sprint performance from baseline to pre-HD60.

#### HD60 (pre-post) differences

**Fig. 3:**
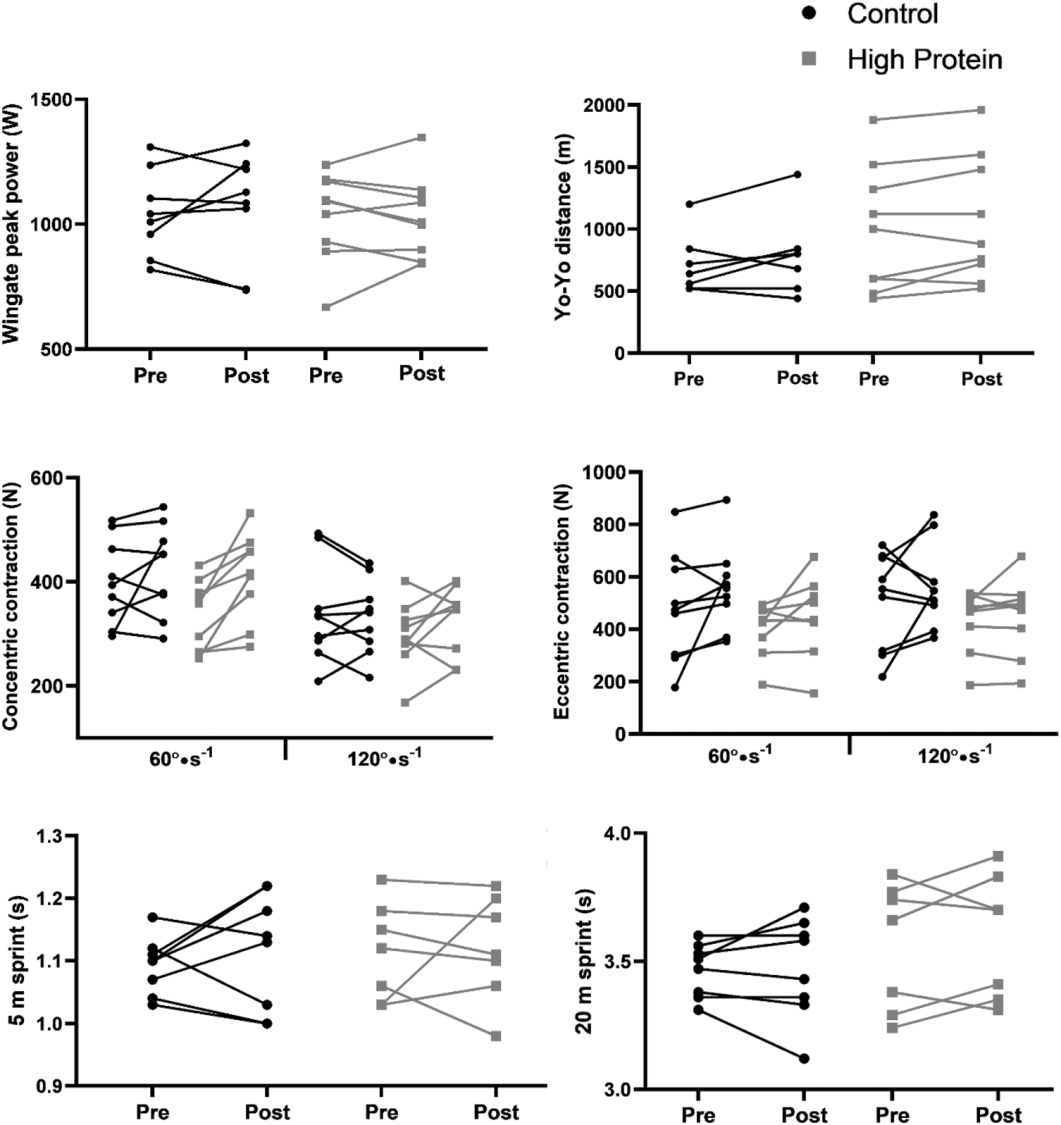
Individual exercise performance measures at pre- and post-energy restriction. Control diet = 0.86 g·d·kgBM^−1^protein; high protein = 1.7 g·d·kgBM^−1^protein; pre = pre 40% energy restriction (HD60); post = post HD60. *Denotes statistically significant change from pre (*p* < 0.05; paired samples *t* test)

Following HD60, there were no significant interactions between time point (pre-post) and dietary condition on any exercise performance measurement. HD60 did not induce any changes in Wingate performance, anaerobic endurance, or sprint performance in either the HP or CON groups, nor were there any between-group differences. Concentric strength at 60°s^−1^ improved during HD60 (*p* = 0.004) in the HP group only. When data from both HP and CON groups were pooled, isokinetic concentric and eccentric strength at 60°s^−1^ significantly improved (*p* = 0.006 and 0.045) during HD60. Individual values for pre-post exercise performance measures are displayed in Fig. 3.

### Menstrual cycle phase

Measurements of serum progesterone concentration were obtained at pre- and post- HD60 in 14 participants. From these data, 11 participants were estimated to be in the follicular phase and 3 in the luteal phase at pre-HD60 (serum progesterone: 2.51 ± 0.38 and 16.45 ± 0.80 ng·L^−1^, respectively), and 12 participants were estimated to be in the follicular phase and 2 in the luteal phase at post-HD60 (serum progesterone: 2.75 ± 0.35 and 18.35 ± 2.20 ng·mL^−1^, respectively). Overall, 9 participants were estimated to be in the follicular phase during both the pre- and post- HD60 DXA measurements.

## Discussion

This study was designed to investigate the impact of increasing dietary protein intake relative to total energy intake on changes in body composition during energy restriction in well-trained females. This study is the first to investigate the impact of protein intake on body composition during an acute period of weight loss in trained females. As expected, total body mass was reduced during 2 weeks of 40% energy restriction. However, we report no discernible differences in changes in body composition between groups consuming a hypocaloric diet containing either 35% or 15% of total energy intake from protein. Exercise performance was not impaired by energy restriction and only concentric strength was affected by protein intake. Overall, our findings suggest that increasing protein intake to 35% of total energy intake does not induce LBM change, independently, or relative to reducing protein intake to 15% of total energy intake in trained females during 2 weeks of diet-induced weight loss. These results are in contrast with those previously reported in trained males (Mettler et al. 2010).

Any sex differences in body composition changes with increased relative dietary protein composition during weight loss may be due to a number of factors. Various individual pre-existing characteristics, including body composition, may play a large determining role in weight loss and body composition succeeding dietary and/or exercise intervention. In this regard, Forbes (2000) and Heymsfield et al. (2011) noted that leaner individuals have greater susceptibility to LBM loss during weight loss, relative to those with higher body fat mass. As such, it may be expected that males and females respond differently to energy restriction, with regards to changes in LBM, owing to notable sex differences in body composition. These findings (Forbes 2000; Heymsfield et al. 2011) may partly explain the discrepancies between past and present findings, whereby severe (~ 40%) energy restriction induced significant diminishment of LBM in well-trained males (Mettler et al. 2010), but not in well-trained females. These inconsistencies between males and females regarding LBM change may have been somewhat predetermined by their baseline body fat (~ 17% and ~ 27%, respectively), which perhaps limited a substantial loss of LBM in females. Accordingly, higher adiposity in females may protect against energy restriction-induced loss of LBM in the short-term, and perhaps longer periods of energy restriction (> 2 weeks) are needed for a measurable change in LBM to be detected in females. Noteworthy, it appears that as baseline body fat increases (40–50%), the protective effect of body fat on LBM loss is diluted, with untrained females losing up to ~ 6% LBM in 10–20 weeks (Gordon et al. 2008; Layman et al. 2003, 2005; Leidy et al. 2007; Mateo-Gallego et al. 2017; Noakes et al. 2005). Thus, it seems feasible that baseline body composition may be a stronger prerequisite to changes in LBM during weight loss in lean trained individuals than in overweight and untrained populations.

Changes in LBM during weight loss may be further influenced by absolute protein intake. LBM was preserved in trained males during energy restriction by increasing protein intake to 2.3 g·d·kgBM^−1^ (Mettler et al. 2010). This increase was achieved by increasing relative protein intake from 15 to 35% of total energy intake. However, in our present study, increasing protein intake from 15 to 35% of total energy intake resulted in no increase in absolute protein intake relative to BM. These females had a substantially lower habitual energy intake compared to the males (Mettler et al. 2010). Thus, increasing the percentage of energy from protein during HD60 did not change absolute protein intake in the current study (1.7 g·d·kgBM). Current recommendations state that energy-deficient athletes may require up to 2.4 g·d·kgBM^−1^ of protein to preserve or increase LBM (Witard et al. 2019). In the present study, LBM was preserved when protein was consumed at 0.9 and 1.7 g·d·kgBM. Thus, in young trained females, the amount of daily protein required to preserve LBM during energy restriction may be less than previously recommended (Witard et al. 2019), at least during short periods of energy restriction. To elucidate the role of dietary protein in LBM change in energy-restricted, young, trained females, longer-term intervention studies may be required.

The change in dietary protein intake from habitual protein intake may modify LBM change during energy restriction. Participants in Mettler’s study habitually consumed 1.6 g·d·kgBM^−1^ of protein and, during energy restriction, the high protein group consumed 2.4 g·d·kgBM^−1^, which attenuated LBM loss. However, protein intake in the control group was reduced to 1.0 g·d·kgBM^−1^, resulting in loss of LBM (Mettler et al. 2010). Therefore, it is not possible to ascertain whether the statistical difference in LBM change was attributed to an increase in protein intake in the high protein group, or a reduction in protein intake in the control group. In the present study, we observed the effects of maintained versus reduced absolute protein intake and did not report between-group differences. Thus, our study suggests that changes in LBM are likely attributed to increased, rather than decreased, protein intake, which supports the conclusions of Mettler et al. (2010). Therefore, sufficiently increasing absolute protein intake during energy restriction seems a plausible determinant to LBM change.

Other factors, including the duration and/or degree of energy restriction, may influence changes in body composition. In the present study, LBM was not significantly reduced with 2 weeks of 40% energy restriction, with the HP and CON groups losing only 100 g and 300 g of LBM, respectively. Nevertheless, the difference in LBM change between groups may become more pronounced with longer periods of energy restriction. It is conceivable that we failed to detect a statistically significant reduction in LBM due to the short duration of the energy restriction. However, it has previously been demonstrated that 2 weeks of energy restriction was sufficient to induce LBM loss in males (Mettler et al. 2010). Thus, the duration of energy restriction per se does not seem to explain our results. Potentially, the severity of energy restriction is a stronger mediator of LBM change than the duration of energy restriction (Garthe et al. 2011). However, the reduction in energy restriction was identical between past (Mettler et al. 2010) and present studies. Therefore, neither the reduction in energy intake, nor the duration of energy restriction, appear to explain the discrepancies between males (Mettler et al. 2010) and females. Future research exploring these conceivable sex differences in the physiological response to energy restriction are warranted.

Severe energy restriction can, however, be detrimental to various exercise performance parameters, including strength, sprinting, jumping, and sport-specific fitness tests (Fortes et al. 2017; Garthe et al. 2011; Lalia et al. 2018; Ribas et al. 2019). In the present study, exercise performance was maintained and, in the most part, unaffected by protein intake. Although, due to the great degree of intra-participant variability in the exercise protocols utilised, our data may fail to represent true changes in performance. Regardless, meaningful performance change is unlikely to occur over 2 weeks and previous short-term periods (5–14 days) of severe energy restriction did not deteriorate strength, power, aerobic or anaerobic capacity, despite significant weight loss (Durguerian et al. 2016; Mendes et al. 2013; Zachwieja et al. 2001). Short-term energy restriction does not appear to adversely affect exercise performance, although the long-term effects of severe energy restriction using sport or event-specific measurements of performance are poorly understood. Accordingly, future longitudinal studies investigating the impact of long-term energy restriction on exercise performance would be beneficial.

The authors acknowledge that a lack of control for menstrual cycle status may be considered a limitation of the present study. A key consideration when conducting research with premenopausal females is hormonal status, i.e., menstrual cycle phase and use of hormonal contraceptives. However, we did not control menstrual cycle phase, which may have influenced the DXA measurements due to cyclic fluctuations in body water. During the DXA measurements, conducted on days 7, 14, and 28 of the intervention, some of the females were not in the same phase of the menstrual cycle (as estimated by serum progesterone concentration) at each measurement. Furthermore, there was between-participant variation in cycle phase at each DXA measurement, which may have created inter-participant variability in lean body mass change. Nonetheless, studies that have compared body composition measurements across the menstrual cycle have not reported significant differences between menstrual cycle phases (Cumberledge et al. 2018; Hicks et al. 2017). However, endurance- and resistance- based exercise performance may be impaired during the early follicular phase of the menstrual cycle, albeit this response is likely individual and supporting evidence is inconclusive (McNulty et al. 2020). Future studies in females should consider hormonal status and, where feasible, conduct primary measures during the same phase of each menstrual cycle.

## Conclusion

In summary, we demonstrate that short-term severe energy restriction reduced total body mass, in the absence of change to LBM and exercise performance. However, our study failed to show a difference between higher and lower protein intakes with respect to changes in LBM in well-trained females, which conflicts previous research in well-trained males. These data indicate that neither maintaining nor reducing absolute protein intake diminishes LBM during short-term energy restriction in trained females. The impact of protein intake on LBM change during energy restriction warrants investigation in trained females. Furthermore, recommendations for weight loss in trained females should express protein intake relative to body mass, not energy intake.

## Abbreviations

ANOVA: Analysis of variance
BM: Body mass
CON: Control
FM: Fat mass
HD60: Habitual diet at 60% of energy requirements
HD100: Habitual diet at 100% of energy requirements
HP: High protein
LBM: Lean body mass
MPB: Muscle protein breakdown
MPS: Muscle protein synthesis
